# Numerical format and public perception of foreign immigration growth rates

**DOI:** 10.1371/journal.pone.0310382

**Published:** 2024-10-02

**Authors:** Maria Michela Dickson, Giuseppe Espa, Rocco Micciolo, Lucia Savadori

**Affiliations:** 1 University of Padova, Padova, Italy; 2 University of Trento, Trento and Centre of Security and Crime Sciences, University of Trento and the University of Verona, Trento, Italy; 3 University of Trento, Rovereto, Italy; 4 University of Trento, Trento, Italy; Queen Mary University of London School of Business and Management, UNITED KINGDOM OF GREAT BRITAIN AND NORTHERN IRELAND

## Abstract

The study aimed to explore whether the 1-in-X bias is also present in relation to immigration growth rates. We tested this research question on a representative sample of adult residents in Trento, Italy, between March and April 2019. Participants were presented with data comparing the foreign immigrant-to-resident population ratio in Italy for 2001 (1 in 40) and 2011 (1 in 15), using two distinct formats—1-in-X and percentages. They were then asked to express the perceived increase. Baseline measures of several individual-level factors, including cultural worldviews, perceptions of immigration, numeracy, science literacy, and economic literacy, were also collected to explore the potential role of individual differences in influencing the effect of the 1-in-X format on the perceived increase in immigrants. The results confirmed the existence of the 1-in-X bias, demonstrating that the immigration growth rate in the 1-in-X format was perceived as higher than in the percentage format, even after controlling for the effects of the idiosyncratic variables. The results of this study provide insight into how different numerical formats can influence public perceptions of immigration growth rates, offering suggestions to policymakers, communicators, and stakeholders about how the presentation of information can shape public opinion.

## Introduction

We frequently assume that facts and statistics are objective and should have a straightforward impact on public opinion. However, while capable of remarkable intellectual feats, the human mind often navigates the complexities of decision-making using simplifying shortcuts or heuristics. This tendency is well-documented in cognitive psychology, particularly in the study of judgment and decision-making, which has identified specific systematic errors in thinking, known as cognitive biases (e.g., [1–4]). One such cognitive bias is the 1-in-X bias [5].

The 1-in-X bias represents a divergence between intuitive understanding and mathematical reality, illustrating how humans perceive probabilities. Individuals influenced by the 1-in-X bias perceive a statistic as corresponding to a higher probability when framed as “1-in-X” rather than “N-in-NX” or percentages, even though the presentations depict the same probability. For example, a 1 in 200 probability of contracting a disease is perceived to be higher than a 5 in 1000 chance, and 1 in 50 is perceived as higher than 2%. This bias highlights the effect of presentation on cognition, revealing the gap between perceived and true probabilities [5, 6]. While the 1-in-X bias has been established across diverse domains and populations [5–12], its application to the perception of immigration growth rates remains unexplored.

In this study, we aim to fill this gap by exploring whether the 1-in-X bias is also present when related to immigration growth rates. We do so by examining how the 1-in-X presentation format (as opposed to percentages) used to communicate immigration growth rates affects public perceptions of such phenomena. Exploring the impact of the 1-in-X bias on perceptions of immigration growth rates is important because public perceptions can significantly influence immigration policies and social integration efforts [13]. Understanding this bias in the context of immigration can facilitate the development of more effective communication strategies that accurately convey statistical information to the public.

Investigating whether the 1-in-X bias also affects public perceptions of immigration growth rates might yield both theoretical and practical contributions. Demonstrating that this bias also affects perceptions of growth rates, rather than static statistical values (e.g., 1 in 200), can improve our understanding of the 1-in-X bias. Previous research has primarily analyzed the effect of the 1-in-X format on individual statistical values (e.g., 1 in 200). Only one study [12] has explored the 1-in-X bias in relation to perceptions of perceiving changes between two statistical values (e.g., a probability shift from 1 in 200 to 1 in 250). However, no prior research has investigated the 1-in-X bias in the context of changes between two values, comparing a 1-in-X format with a percentage format. Thus, the present study provides a theoretical contribution to the literature by generalizing the 1-in-X effect to include the comparison between these two formats in the context of perceived changes.

Addressing whether the 1-in-X bias also impacts the perception of immigration growth rates might also have practical implications because public perceptions of immigration rates can impact citizens’ attitudes and behaviors toward immigrants [14] and influence political voting behavior and influence political vote [13, 15].

Citizens’ perception of immigration growth rates have been explored in various studies. One significant finding is that citizens’ perceptions of immigration levels are influenced more by the growth of local immigrant populations over time rather than their absolute size [16]. This suggests that the growth rate is more likely to capture citizens’ attention and influence their perceptions of immigration levels. In the present study, we therefore focused on the immigration growth rate and not merely the number of immigrants in a community. More generally, research has uncovered a tendency to overestimate the demographic size of minority populations in both the United States and Europe (e.g., [13, 17]). Unsurprisingly, individuals who significantly overestimate the proportion of immigrants are also more likely to oppose immigration. This is possibly because they perceive a heightened “threat” and feel that their country is disproportionately targeted compared to similar countries [13]. Although research by [18] has proven that simply offering accurate data about immigration does not significantly alter public opinion, no study has addressed whether presenting information in differing formats, such as the 1-in-X format compared to percentages, could affect how people perceive immigration growth rates.

Immigration growth rates are typically reported to the public in percentage format (e.g., the proportion of immigrants in the United States was 9.22% of the population in 1999 and 15% of the population in 2020). News reports about the foreign immigrant growth rate can strongly impact public sentiment and voting behavior and have far-reaching social, economic, and political implications in relation to public opinion [13, 15]. People may form biased opinions if the information presented is not neutral [19–21]. Although the statistics are accurate and are typically provided by government institutions, the format in which they are reported is dictated by the corresponding news outlet or institution. The same information can be presented in either percentage format (%) or ratio format (1-in-X) without affecting its accuracy, as both report mathematically equivalent values. However, the choice of format is not trivial, as it could influence public opinion and, potentially, public behaviors.

**Fig 1:**
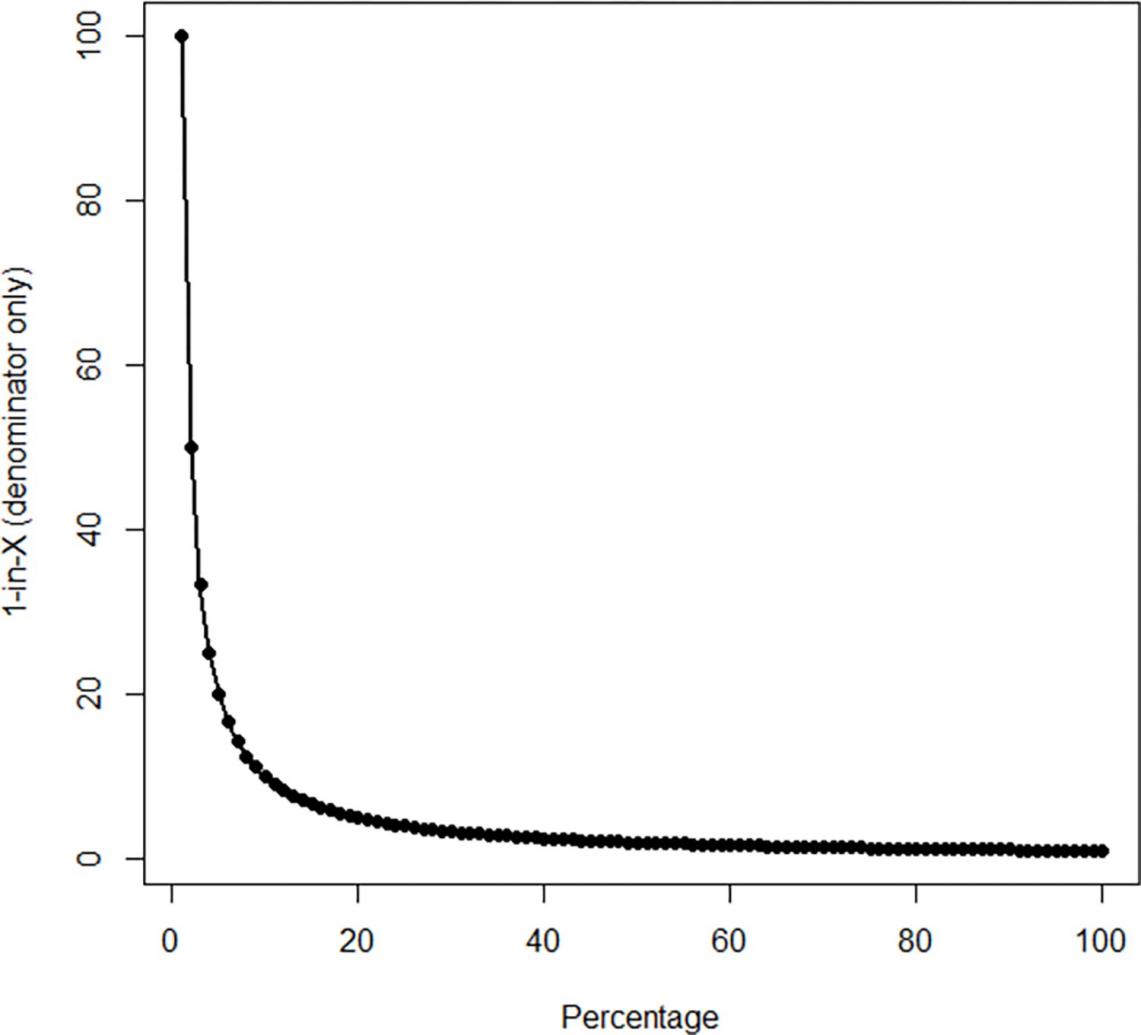
Plot of the relationship between the values of the denominator in the 1-in-X format (y-axis) and the values of the numerator in the percentage format (x-axis).

To explain why the 1-in-X format leads to an overestimation of the perceived frequency, [12] suggested that people discern changes in the rate of occurrence of an event in the 1-in-X format by focusing on the nominal value of the denominator of the ratio, which is the “changing element” (i.e., the numerator “1” does not change). In contrast, people judge changes in the rate of occurrence of an event in other formats, such as percentages or population-based frequencies, by focusing on the nominal value of the numerator of the ratio, which is the “changing element” (i.e., the denominator “100” does not change). Given that the difference in the nominal values between the denominators in the 1-in-X format is greater than the difference in the nominal values between the numerators in the percentage format (as illustrated in Fig 1), people perceive the change as being greater when communicated in the 1-in-X format compared to the percentage format. In other words, the marginal variation of the denominator in the 1-in-X format increases, while the marginal variation of the numerator in the percentage format remains constant. In this study, we hypothesize that presenting immigration growth rates in a 1-in-X format will lead to a higher perceived increase than when presented in percentage terms.

While the 1-in-X bias has been documented as a significant phenomenon in numerous studies, it is important to acknowledge that not all research has found it to be as strong and pervasive as originally hypothesized. For instance, using a sample from the Italian population, [22] did not observe the 1-in-X effect. Similarly, a review by [9] suggests that the effect might be less pronounced than initially believed. The variability in such findings points to the need for further research to explore the conditions under which the 1-in-X effect is more or less pronounced. This could include examining individual differences, including cultural worldviews [23], concerns about immigration [24–26], numeracy [27–30], scientific literacy [31], and economic literacy [32]. These factors were selected for their potential interactions with the 1-in-X effect in relation to peoples’ risk perception and perception of immigration growth rates. We will therefore also explore the potential role of individual differences in influencing the effect of the 1-in-X format on the perceived increase in immigrants.

According to the existing literature, risk perceptions are shaped by cultural worldviews. [23] introduced the cultural cognition theory, which is rooted in Douglas and Wildavsky’s cultural theory of risk [33]. This theory categorizes individuals along two axes related to their principal worldviews: hierarchical versus egalitarian, and individualist versus communitarian. Hierarchical-individualists, who view society through a classist lens and prioritize personal freedom, tend to have lower risk perceptions, display reduced trust in institutional information sources, and exhibit inaccuracies when interpreting numerical data, such as statistics from contingency tables concerning the effectiveness of immigration policies [34–36]. In contrast, egalitarian-communitarians prioritize social equality and the collective good over individual interests. Cultural worldviews not only influence the perception of risks but also guide the interpretation of scientific facts and other risk-related information [37]. Thus, these worldviews may also affect susceptibility to cognitive biases, such as the 1-in-X effect, particularly in relation to immigration growth rates.

Biased information processing can also be the result of a biased self-selection of information [24–26] guided by people’s motivations [38]. Consequently, individuals’ public opinion regarding immigration might influence the 1-in-X format bias. In addition, increased numerical, scientific, and economic skills may mitigate reasoning biases [27–31] and impact perceptions of immigration [39]. Based on this literature, these skills may mitigate the 1-in-X bias in relation to immigration growth rates.

## Methods

### Sample

The Perception of Risks connected to Immigration (PRI) survey was conducted between March 1st and April 30th, 2019, with a representative sample of residents in the city of Trento in Northern Italy [39]. The primary aim of the survey was to evaluate the effects of numerical, scientific, and economic literacy on immigration concerns when controlling for cultural worldviews. The reference population for the survey consisted of adult citizens between the ages of 18 and 80 of both genders residing in the city, selected from the municipal register updated on January 1st, 2019. A population total of 90,051 individuals was considered, excluding the homeless and nomads. A stratified sampling design was implemented. The population was stratified into non-overlapping homogeneous groups (strata) based on gender (female, male), date of birth (from 18 to 35 years, from 36 to 55 years, and from 56 to 80 years), and district of residence (originally 12 districts, aggregated in three macro-areas basing on socio-demographic features), resulting in 18 strata. A random sample of 2,008 units was selected from the population, following the aforementioned stratification (see S1 Table for absolute frequencies and percentages of the population and the selected sample) by using the R package sampling [40].

The allocation of the sample in each stratum was proportional to the stratum size, with a minimum number of units in each stratification cell *n_h_* = 5 and a maximum *n_h_* = *N_h_*, forcing the allocation to be *n_h_*= 5 when *n_h_*<5, and censusing the stratum when *n_h_* = 5. The selection of the units in each stratum occurred by means of simple random sampling without replacement, with an inclusion probability for the *h − th* stratum equal to $\pi_{h}=\frac{n_{h}}{N_{h}}$.

The survey was conducted using structured questionnaires and administered to the sample units using CAWI/CATI methodologies. After a written invitation was delivered by mail, the sampled units were invited to connect to the statistical survey web platform Limesurvey to complete the questionnaires anonymously (CAWI). Furthermore, for those who were unable to compile it independently, a service was made available to execute guided compilation with an operator (CATI). The response rate to the survey was equal to 27% (551 units). The set of respondent units was nearly balanced for gender, age groups, and macro-area of residence (Table 1 reports the absolute frequencies and percentages).

**Table 1:** Response rate (absolute frequencies and percentages) stratified by gender (female/male), age groups (18–35, 36–55, and 56–80 years), and macro-area of residence (South-West, Center-North, North-East).

		Absolute frequency	%
	Female	270	49.0
**Gender**	Male	281	51.0
	Total	551	100
	18–35	108	19.6
**Age**	36–55	188	34.1
	56–80	255	46.3
	Total	551	100
	South–West	178	32.3
**Macro-area**	Center–North	234	42.5
	North–East	139	25.2
	Total	551	100

Collected data were corrected for non-responses [41] to produce unbiased estimates. A calibration estimator was implemented [42], forcing the calibration on the three stratification variables’ totals. The implementation was conducted through the R package survey [43].

### Procedures and materials

The PRI questionnaire was composed of several sections, including one in which all participants were given the following instructions: “We would like you to read some information about immigration and provide your opinion on it.” Participants were then given information in one of two experimental formats (1-in-X or percentage) based on their random assignment to one of the two groups using the following words:

*“Consider the following data: “In 2001, the rate of foreign immigrants relative to the resident population was [1 per 40 inhabitants; 2.5%]; in 2011, this rate increased to [1 per 15 inhabitants; 6.6%]*”.

We did not specify whether the data pertained to Trento, the region, or Italy as a whole, given that the information represented a rate as opposed to absolute numbers. The dependent variable was the Perceived increase in the immigration rate. Participants were asked to complete the following sentence: *“According to you*, *this increase is*:*”*. They had to do so by choosing options from a 5-level Likert scale: very low (1), low (2), neither high nor low (3), high (4), very high (5). A sixth option, namely “don’t know/don’t answer,” was also listed and was chosen by 17 units (3% of total respondents). These units were not considered in the analysis; thus, the final sample used was composed of 534 units. The information on immigration statistics provided to participants was based on actual statistics from the Italian National Institute of Statistics [44, 45].

### Idiosyncratic variables

A set of baseline measures were collected at the beginning of the survey. These measures served as control variables, helping to adjust for some potential idiosyncratic factors that may have influenced the results. By including these variables in our analysis, we were able to enhance the accuracy of our interpretation of the results and determine the impact of the independent variables.

### Cultural worldviews

The short version of the cultural worldviews scale developed by [46], consisting of 12 statements designed for bipolar dimensions, was used to measure cultural worldviews (see S2 Table). Participants were asked to rate their level of agreement or disagreement on a five-point scale, with “1” meaning “completely agree” and “5” meaning “completely disagree.” The responses were treated as numeric and averaged to create a composite score, with higher values indicating a stronger adherence to the hierarchical-individualist worldview, such as a conservative profile. By averaging all the responses, the strength of the hierarchical-individualist worldview was obtained (*μ* = 2.63; *SD* = 0.45) by means of a composite overall index (*α* = 0.73).

### Perception of immigration

To assess participants’ perceptions of immigration, a set of 13 items was used (see [39]). Ten statements were derived from the General Social Survey 1972–2014 [47], while the other three questions concerned risk perception (see S3 Table). The first 12 items explored the participants’ opinions about the impact of immigrants on Italian culture, economy, crime, rights, public spending, refugees, and the overall risk and benefit that immigration posed to Italian society. Respondents indicated their agreement or disagreement on a 5-point scale, with “1” indicating “completely agree/not at all risky/beneficial” and “5” indicating “completely disagree/extremely risky/not at all beneficial.” The 13th item consisted of an emotion-related question (*“Thinking about the immigration phenomenon in Italy makes you feel __________ emotions”*) to which participants responded on a 5-point scale, with “1” indicating “very negative” and “5” indicating “very positive.” All but the last item were reverse-coded so that higher values corresponded to more favorable perceptions of immigration. Responses were grouped in a composite average index (*α* = 0.94; *μ* = 3.16; *SD* = 0.75), reflecting the degree of tolerance toward immigration. We checked for idiosyncratic factors by using principal component analysis, which demonstrated that the principal component explains about 60% of the total variability and that the factor loadings are substantially equal to each other. Notably, the mean of the 13 items had a correlation index equal to 0.98 with the score given by the first principal component (see for details, [39] and response to reviewers available on the journal webpage in the “Peer review” section). Therefore, we decided to proceed with the use of items’ mean, considering it more informative.

### Numeracy

The numerical skills of each respondent were evaluated using a 5-item numeracy test (see [39]) adapted from [48]. Each question presented four options, but only one was correct (see S4 Table). The individual numeracy score was calculated by summing the number of correct answers, with a higher score indicating a higher level of numerical ability (*μ* = 3.22; *SD* = 1.11).

### Scientific literacy

The objective scientific knowledge of each participant was measured using 10 true-false questions from the Science and Engineering Indicators [49]. The items concerned clinical, biological, and technological factors (see S5 Table). A composite score was calculated by adding up the number of correct answers, with higher values indicating a greater level of objective scientific literacy (*μ* = 8.39; *SD* = 1.70)

### Economic literacy

A set of 12 multiple-choice questions from the Test of Economic Knowledge [50] was used to assess economic literacy (see [39]). Items were selected to be sufficiently easy [50] and representative of different test contents (see S6 Table). The individual index was calculated based on the number of correct answers given (*μ* = 8.91; *SD* = 2.67).

## Data analysis strategy

The dependent variable was the Perceived increase in the immigration rate relative to the resident population; it was classified as either “high” or “very high.” More precisely, the variable was ranked on a 5-level Likert scale as very low (1), low (2), neither high nor low (3), high (4), or very high (5). The decision about this demarcation (namely, categorizing respondents reporting the first three levels separately from respondents reporting the last two) is based on the analysis of the unadjusted and adjusted odds ratios, with the associated 95% confidence intervals. The results revealed that the chosen cutoff maximized the effect size. The entire analysis and results are reported in S1 Appendix. The treatment variable was the Format, which was at two levels: 1-in-X or percentage. Cultural worldviews, perceptions of immigration, numeracy, scientific literacy, economic literacy, age, and gender were considered potential idiosyncratic variables.

Initially, a logistic regression model with main effects in which the dependent variable was regressed on all the idiosyncratic variables was estimated. Successively, the effect of the Format was inserted in the model. Finally, a third logistic regression, in which all the interactions of the idiosyncratic variables with the Format were considered, was estimated.

The odds ratio (i.e., the exponential of the regression coefficient for the Format) was considered a measure of *effect size*. The significance of the effect was determined through a likelihood ratio test (LRT).

## Results and discussion

Table 2 presents the full distribution for the 5-level Likert scale regarding the Perceived increase in the immigration rate for the two formats.

**Table 2:** Distribution (absolute frequencies and, in parentheses, row percentages) for all 5 levels on the Likert scale and the two experimental formats.

	Perceived increase in the immigration rate					
**Format**	Very low	Low	Neither high nor low	High	Very high	**Total**
1-in-X	2 (0.7)	14 (5.1)	49 (17.7)	121 (43.7)	91 (32.9)	277 (51.9)
Percentage	2 (0.8)	28 (10.9)	88 (34.2)	90 (35.0)	49 (19.1)	257 (48.1)
**Total**	4 (0.7)	42 (7.9)	137 (25.7)	211 (39.5)	140 (26.2)	534 (100)

Overall, approximately two thirds of the participants (351 out of 534, 65.7%) perceived the increase in the immigration rate as “high” or “very high.” However, there was a significant difference in this perception between the two experimental groups: When the information was presented in percentage, 54.1% of the participants (139 out of 257) considered the increase to be “high” or “very high,” while when the information was presented in the 1-in-X format, this percentage was 76.5% (212 out of 277). This resulted in a change in the odds of perceiving the increase as “high” or “very high” from 1.18 (139/118) to 3.26 (212/65), yielding an odds ratio of 2.77. As expected, the impact of the Format was significant, as evidenced by both Fisher’s exact test (*p* < 0.001) and a likelihood ratio test (Chi-square = 30.1; *df* = 1; *p* < 0.001). The true odds ratio was estimated to have a 95% confidence interval (C.I.) between 1.92 and 4.03. This indicates that the odds of perceiving the increase in the immigration rate as “high” or “very high” were 2 to 4 times greater when presented in the 1-in-X format.

A set of logistic regressions was performed to assess whether the observed effect could be accounted for by potential idiosyncratic variables. These regression models were used to estimate the odds ratio of the Format, using an approach involving three steps.

Firstly, a logistic regression containing only the main effects, considering all the potential idiosyncratic variables, was performed (model 1). In this case, only the variable Perception of immigration was significant (*p* < 0.001). In the second step, the effect of the Format was inserted in the model to control for the other variables (model 2). The effect was clearly present and was significant (LRT = 42.742; *df* = 1; *p* < 0.001). Finally, the interactions between all the idiosyncratic variables and the Format were considered in a third model (model 3). Here, the LRT discards all interactions (LRT = 7.417; *df* = 8; *p*–value = 0.492). The estimated coefficients of model 2 are shown in Table 3. To ensure the completeness of the analysis, S2 and S3 Appendices report the results for, respectively, ordinal logistic regression and linear regression models, following the same approach in three steps presented here.

**Table 3:** Logistic regression results for model 2 which included as independent variables all the potential idiosyncratic variables and the effect of Format on the Perceived increase in the immigration rate relative to the resident population as “high” or “very high”. When this effect was inserted, controlling for the other variables, the likelihood ratio test was significant (LRT = 42.742; *df* = 1; *p* < 0.001).

Variables	Estimate	Std. error	*z* value	*p*–value
(Intercept)	4.307	0.928	4.642	*p*<0.001
Gender				
Male vs Female	0.153	0.221	0.694	0.487
Age				
36–55 *vs*. 18–35	0.286	0.309	0.926	0.354
56–80 *vs*. 18–35	–0.199	0.289	–0.691	0.490
Numeracy	–0.171	0.116	–1.467	0.142
Economic literacy	0.053	0.053	0.991	0.322
Scientific literacy	0.148	0.085	1.740	0.082
Cultural worldviews				
HI *vs*. EC	–0.136	0.238	–0.572	0.568
Perception of immigration	–1.670	0.199	–8.384	*p*<0.001
Format				
1-in-X *vs*. Percentage	1.393	0.223	6.261	*p*<0.001

The results indicate that the format effect (1-in-X vs. percentages) was significant in support of our hypothesis. The increase in the immigration rate communicated in the 1-in-X format was perceived as higher than the same increase communicated in percentages. In addition, the effect of the 1-in-X format bias was not modified by any of the idiosyncratic variables. It remained not only significant (*p* < 0.001) but also relevant (the odds ratio is 4.03).

Beyond not influencing the impact of the 1-in-X bias, the idiosyncratic variables were also not associated with the Perceived increase in the immigration rate, with the exception of the Perception of immigration, which was negatively associated to it (with a coefficient that is, in absolute value, higher than that of the Format). Individuals holding a more negative view of immigration perceived the immigration growth statistic (irrespective of its numerical format) as higher than those individuals holding a more positive view of immigration.

Overall, approximately two thirds of the participants (351 out of 534, 65.7%) perceived the increase in the immigration rate as “high” or “very high.”

**Fig 2:**
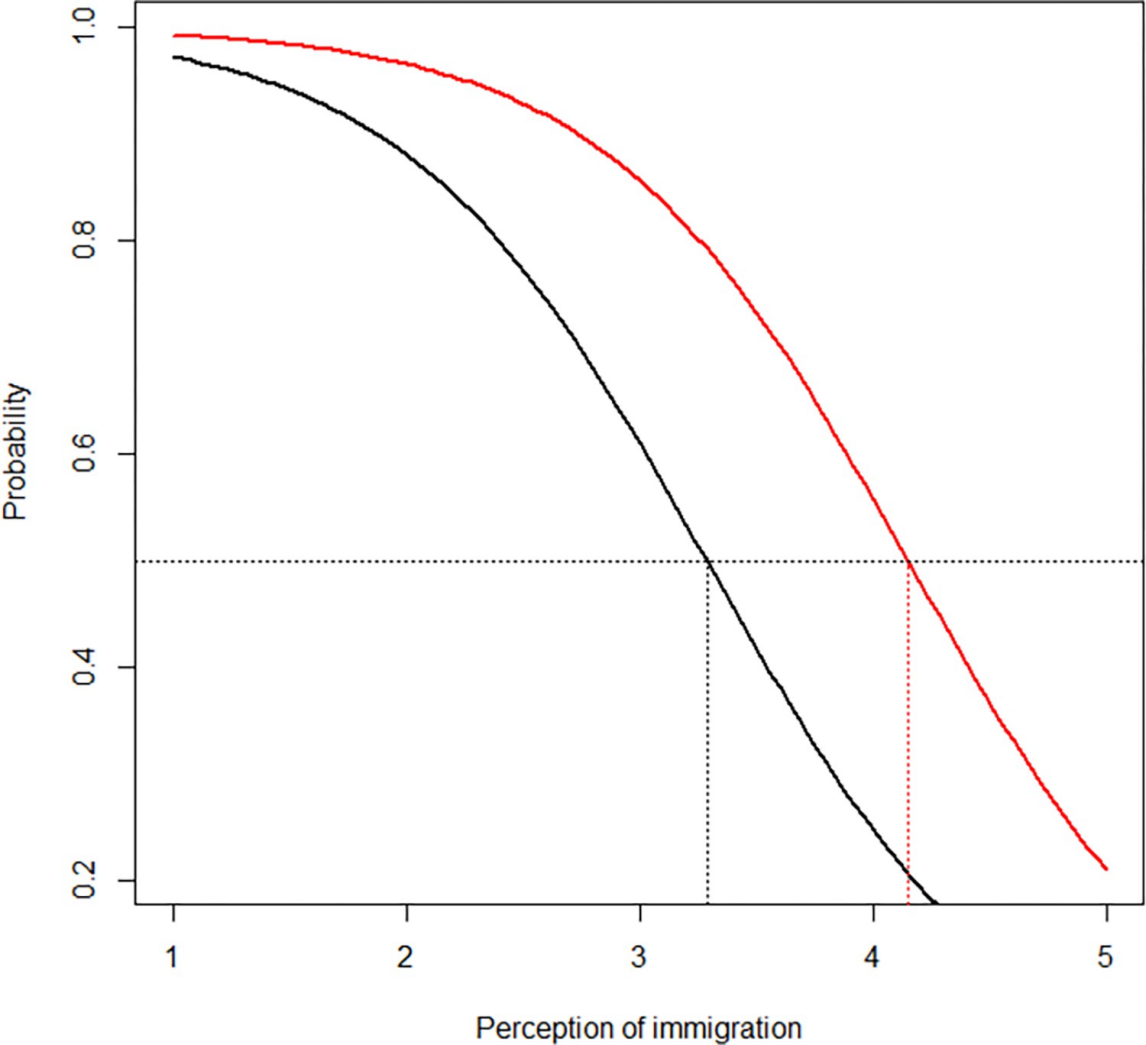
Estimated probabilities of perceiving the increase in the foreign immigration rate as “high” or “very high,” based on the Perception of immigration and depending on the Format. The red curve represents the 1-in-X format, and the black curve represents the percentage format.

Fig 2 illustrates the estimated probabilities of perceiving the increase in the foreign immigration rate as “high” or “very high,” based on Perception of immigration and on the format used. The red curve, which represents the 1-in-X group, consistently lies above the black curve, representing the percentage format. Therefore, with the Perception of immigration held constant, the 1-in-X format is linked to a higher likelihood of perceiving the increase in the immigration rate as “high” or “very high,” compared to the percentage format. Notably, the gap between the two lines, indicative of the 1-in-X format bias, remains uniform across most of the values of the *x* variable, namely Perception of immigration. This suggests that the bias is equally present among those who oppose immigration and those who do not. Furthermore, Fig 2 also highlights the significant influence of opinions regarding immigration: Independently of the Format, statistics are perceived as more extreme among individuals with more negative views of immigration.

For instance, among individuals with a high tolerance for immigration (score of 4 on the Perception of immigration), about 25% perceived the increase in the immigration rate as “high” or “very high” when presented in percentage format (the black curve). This proportion rose to 56% with the 1-in-X format (the red curve). Thus, individual attitudes toward foreign immigration affect perceptions of increases in immigration rates, regardless of the format used. Despite observing the same objective rates, individuals who feel more favorably toward immigration assessed it as occurring at a lower rate than those who feel less favorably.

The results obtained are essentially robust in the sense that they do not depend on the choice of the cutoff or even the type of statistical analysis performed (logistic regression or ordinal logistic regression), as can be seen in S1 and S2 Appendices. Transforming the odds ratios to Cohen’s d [51] employing the approach of [52], the values obtained for the Cohen’s effect size ranged from 0.46 (when the odds ratio was 2.1) to 0.69 (when the odds ratio was 3.1). A similar result was obtained when the Perceived increase in the immigration rate was considered as a continuous variable; in this case, Cohen’s d effect size was 0.46 when comparing the mean values of the Perceived increase in the immigration rate in the two scenarios and increased to 0.59 when comparing the corresponding adjusted difference, after accounting for the effect of selected idiosyncratic variables (see S3 Appendix).

## Conclusions

### Theoretical and practical implications

The results of this study have uncovered relevant insights and several important theoretical and practical implications. Firstly, the results confirm the 1-in-X bias, indicating that the format in which information about the increase in the rate of foreign immigration is presented can significantly impact individuals’ perceptions of this increase. Approximately two thirds of the participants perceived the increase in the rate of immigrants as “high” or “very high,” but this perception was influenced by the format in which the information was presented. When the information was presented in a 1-in-X format, as opposed to a percentage format, there was a significant increase in the odds of perceiving the increase as “high” or “very high.” The odds of perceiving a “high” or “very high” increase in the rate of immigration were estimated to be 2 to 4 times greater when presented in the 1-in-X format compared to percentages. This shows that changes in values presented in the 1-in-X format perceived as higher than mathematically equivalent changes presented in percentages.

These findings expand upon the previous research, which has primarily focused on examining the impact of the 1-in-X format on a single value statistic [5] by exploring its effects on changes between two values. This study confirms that the effect of the 1-in-X format bias can be generalized to changes expressed in percentages, not merely ratios [12]. This provides new insights into the impact of the numerical format on information perception. This highlights the importance of considering the potential impact of the presentation format on information perception, especially when communicating sensitive or controversial information, such as immigration rates. While our findings highlight the potential dangers of the 1-in-X format in inflating risk perception, thereby potentially skewing public opinion and decision-making, there are contexts in which this effect might be ethically and strategically employed to the public’s benefit. In situations in which the public significantly underestimates a risk, leading to insufficient protective measures or apathy, the 1-in-X format could serve as a valuable tool to correct such misperceptions. For example, in public health campaigns where the risk of not vaccinating children against preventable diseases is underestimated, presenting risk information in a 1-in-X format could more effectively convey the urgency and significance of the risks involved, thus encouraging more protective behaviors. However, the ethical implications of employing this format must be carefully weighed. The deliberate manipulation of risk perception, even with the intention of promoting public good, raises ethical questions regarding autonomy, informed consent, and the potential for creating undue anxiety or panic. Thus, communication professionals and policymakers should consider not only the psychological impact of different numerical formats on risk perception but also the moral responsibilities inherent in their choice of communication strategies. The ethical use of the 1-in-X format requires a delicate balance between achieving public health and safety objectives and respecting the public’s right to receive information in a manner that is both clear and non-manipulative.

In relation to the reasons why the 1-in-X format distorts the perception of quantities, one explanation suggests a cognitive mechanism [12]. As illustrated in Fig 1, as the risk level increases, the change in the numerator of the percentage format is lower than the change in the denominator in the 1-in-X format. For example, a 2% to 5% risk level increase corresponds to a 3-unit increase (from 2 to 5) in the numerator. Conversely, a mathematically equivalent risk level increase from 1 in 50 to 1 in 20 corresponds to a 30-unit decrease in the denominator. Notably, the change in the percentage format (i.e., 3 units) is lower than the change in the 1-in-X format (i.e., 30 units). This difference between the percentage format and the 1-in-X format is believed to result in a greater perceived increase in risk when expressed in the 1-in-X format. In our study, the increase in the rate presented in a 1-in-X format likely created the illusion that the difference between the two values was larger because the denominator of the 1-in-X ratio changed more rapidly than the numerator of the percentage ratio. In other words, presenting immigration growth statistics as a ratio (1-in-X) may have engendered the impression that the increase in immigration was large relative to the total population. On the other hand, presenting the same statistics as a percentage may create the impression that the increase is relatively smaller and potentially less relevant.

The results, however, can also be explained by an “affective” account that posits that emotional responses significantly impact how individuals perceive and evaluate risks [53, 54]. The affect heuristic, as discussed by [54, 55], posits that emotional responses significantly impact how individuals perceive and evaluate risks. This concept is supported by studies such as that by [53], which demonstrated that a frequency format (e.g., “Of every 100 patients, ten are estimated to commit an act of violence”) leads to a perception of greater danger than a statistically equivalent expression as a percentage (e.g., “A 10% chance of committing violence”). The use of frequencies and “1” in the numerator in the 1-in-X format can evoke a stronger emotional response compared to more abstract numerical formats such as percentages, thereby amplifying the perceived risk. Research suggests that a single identified victim elicits more empathy than a group of victims [56], and people display diminished sensitivity to the value of life as the number of victims increases [57]. In the context of the 1-in-X bias, the emotional weight of the “1” in the numerator might have amplified risk judgments more than the abstract nature of the percentages. For instance, conveying risk as “1 in 100” should make the scenario more emotionally charged for individuals, as they can more easily imagine or empathize with a single, identifiable case.

The findings of our study corroborate the presence of the 1-in-X bias. However, the present study was not structured to differentiate between the cognitive and affective explanations for this bias. As a result, our research does not provide evidence favoring one explanation over the other. Future studies should be designed to specifically disentangle these two explanations, thereby providing a more detailed understanding of the underlying mechanisms behind the 1-in-X bias.

The study results also suggest that, not surprisingly, individual attitudes toward foreign immigration influenced perceptions of increases in immigration rates. Individuals who had a higher level of tolerance toward immigration perceived the registered change in the rate of immigration as less “high” or “very high” compared to those with lower levels of tolerance. This underscores the importance of considering the influence of personal attitudes on information perception. As suggested by the literature on motivated reasoning [58], the same objective facts might be interpreted and perceived differently based on the lens of one’s opinion. This highlights the need for individuals to be aware of their own biases and to seek out information from multiple sources to gain a more balanced and accurate understanding of a particular issue.

More importantly, our study expands upon the findings of previous research regarding the 1-in-X bias by testing its generalizability to the general population. Unlike most previous studies, which were conducted on convenience samples or on selected populations (e.g., pregnant women), our study tested the bias in a representative sample of the population, varying in idiosyncratic characteristics. The results revealed that the presentation format exerted a significant impact even after controlling for individual differences such as numerical, scientific, and economic skills; personal attitudes toward immigration; and cultural worldviews. This suggests that contrary to our research question, the impact of the presentation format is a robust effect and can be generalizable to a wide range of populations. The fact that none of the individual factors significantly influenced susceptibility to 1-in-X bias presents a twofold challenge. On the one hand, it diminishes the prospect of effectively mitigating this bias through de-biasing interventions. On the other hand, it raises concerns about the potential for the malicious exploitation of this communication technique to influence public opinion and behavior, including voting decisions. The results of the study suggest that improving public education does not necessarily reduce the prevalence of this cognitive bias, as it affects individuals regardless of their levels of numerical, scientific, and economic literacy. Similarly, efforts to decrease susceptibility to cognitive bias by addressing negative stereotypes toward immigrants or changing cultural worldviews may prove ineffective. The impact of the 1-in-X cognitive bias transcends these demographic and attitudinal divides, affecting both those who view immigration negatively and those who view it positively and affecting both hierarchical-individualists and egalitarian-communitarians. The pervasive nature of this cognitive bias underscores the importance of understanding the mechanisms underlying 1-in-X bias and developing effective strategies to prevent it.

The choice of a numerical format should be guided by the goals of the communication and the target audience, as well as an understanding of how different formats may be received and interpreted. The cognitive 1-in-X bias, indeed, can fuel existing misperceptions. These practical implications are important. Indeed, several studies have found that citizens tend to overestimate the number of foreign-born residents in their countries [18, 20], and this phenomenon can influence electoral choices, as evidenced during the Brexit debate [59] and the 2016 U.S. presidential election [60]. Moreover, far-right parties in countries such as Austria, France, the Netherlands, and Switzerland have garnered significant increases in support precisely by adopting strongly anti-immigration positions [61, 62].

### Limitations and future directions

Regarding the limitations and directions for future research, we acknowledge a methodological limitation concerning the measurement scales employed in this study. Our study used several questions to measure participants’ perceptions of immigration (see S3 Table). An inconsistency was noted in the response options across the scales, particularly in how the scales positioned the more positive (e.g., “extremely agree,” “very positive”) and negative (e.g., “extremely disagree,” “very negative”) poles. This inconsistency may have generated some confusion among the respondents, potentially affecting how they interpreted and answered the questions. Such variations in the scale’s direction could affect our results’ reliability and validity. While not invalidating our results, it is important to consider this when interpreting the data. Future studies could benefit from using more uniform response scales to ensure consistency in participants’ understanding and responses. Moreover, future studies should increase the understanding of the mechanism behind the 1-in-X cognitive bias by directly testing the two proposed explanations (the cognitive and affective explanations).

Further clarification is also necessary regarding the applicability of our results to varying probability values. As illustrated in Fig 1, the non-linear, convex function diminishes at the margins, indicating that as the risk level increases from 0 to 0.1 (i.e., x < 10), the corresponding denominator in the 1-in-X format decreases sharply. Conversely, for probability values greater than 0.1 (i.e., *x* ≥ 10), such as an increase from 10% to 20%, the corresponding denominator in the 1-in-X format declines more gradually, demonstrating a slowdown in marginal variation. This observation produces a testable hypothesis for future research: The 1-in-X effect might be diminished or even reversed for values exceeding 10%. For instance, an increase in the numerator from 10% to 20% is likely to be perceived as more significant than a decrease in the denominator from 1 in 10 to 1 in 5. Notably, [12] observed that the 1-in-X bias decreases as probability values surpass 10%, although an inversion of the bias was not observed. These findings suggest that the cognitive explanation of the 1-in-X bias may coexist with an affective explanation, wherein the numeral “1” elicits stronger emotional responses.

Moreover, it is important to emphasize that practical considerations drove our selection of immigration rate data for 2001 and 2011: These were the years for which we had access to accurate statistics [44, 45]. The population census is conducted every ten years, and since our data collection occurred in 2019, the most recent available data were from 2011. However, it should be noted that some participants, particularly those with a hierarchical-individualist worldview, might have perceived this information as psychologically distant, potentially reducing their perceived risk or the influence of the 1-in-X bias. Despite this consideration, our results indicated that respondents’ worldviews did not significantly affect their perceptions of the increase in the immigration rate, contrary to initial assumptions.

Finally, it is important to note that this study was not preregistered. The absence of preregistration may raise concerns regarding the exploratory nature of certain analyses and the formation of post hoc hypotheses. While the study’s hypotheses were developed based on existing literature and theoretical frameworks, and the analyses were conducted with a commitment to scientific integrity, the lack of preregistration represents a limitation. Recognizing the benefits of preregistration in enhancing research transparency and reproducibility, we suggest incorporating this practice in future studies to further strengthen the validity and reliability of the findings.

## General conclusions

The format in which the information is presented impacts the perception of the rate of foreign immigration growth. The results indicate that the numerical format (1-in-X ratios or percentages) in which the increase in the rate of immigration is presented can significantly impact how individuals perceive the information. This highlights the importance of considering the presentation format when communicating data or information to the public, as it can shape the general public opinion. These results have important implications for policymakers, communication professionals, and researchers. They emphasize the need to carefully consider the information’s presentation format and cognitive biases’ role in shaping public perception and decision-making.

## Supporting information

S1 Table Reference population (absolute frequencies and percentages) and selected sample (absolute frequencies and percentages), both stratified by gender (female/male), age groups (18–35, 36–55, and 56–80 years), and macro-area of residence (South-West, Center-North, North-East). (DOCX)

S2 Table Items used in the survey to measure cultural worldviews. (DOCX)

S3 Table Items used in the survey to measure the perception of immigration. (DOCX)

S4 Table Items used in the survey to measure numeracy. (DOCX)

S5 Table Items used in the survey to measure science literacy. (DOCX)

S6 Table Items used in the survey to measure economic literacy. (DOCX)

S1 Appendix Choice of the cutoff. (DOCX)

S2 Appendix Ordinal logistic regression. (DOCX)

S3 Appendix Linear regression. (DOCX)

S1 Data (CSV)
