# Supplementary material for: Numerical format and public perception of foreign immigration growth rates
Source: PLoS One. 2024 Oct 2;19(10):e0310382. doi: 10.1371/journal.pone.0310382 (PMC11446429; doi:10.1371/journal.pone.0310382)
Supplement: S1 Table — (DOCX) [file pone.0310382.s001.docx]

# Appendix S1

**Table S1.** Reference population (absolute frequencies and percentages) and selected sample (absolute frequencies and percentages), both stratified by gender(female/male), age groups (18-35, 36-55, and 56-80 years), and macro-area of residence (South-West, Center-North, North-East).

|  |  | **Population** | | **Sample** | |
| --- | --- | --- | --- | --- | --- |
|  |  | **Absolute frequency** | **%** | **Absolute frequency** | **%** |
|  | Female | 46,261 | 51.4 | 1,031 | 51.3 |
| **Gender** | Male | 43,790 | 48.6 | 977 | 48.7 |
|  | Total | 90,051 | 100 | 2,008 | 100 |
|  |  |  |  |  |  |
|  | 18 – 35 | 23,678 | 26.3 | 530 | 26.4 |
| **Age** | 36 – 55 | 33,555 | 44.8 | 747 | 37.2 |
|  | 56 – 80 | 32,818 | 31.1 | 731 | 36.4 |
|  | Total | 90,051 | 100 | 2,008 | 100 |
|  |  |  |  |  |  |
|  | South – West | 28,028 | 31.1 | 625 | 31.1 |
| **Macro-area** | Center – North | 40,359 | 44.8 | 900 | 44.8 |
|  | North – East | 21,664 | 24.1 | 483 | 24.1 |
|  | Total | 90,051 | 100 | 2,008 | 100 |
