# Supplementary material for: Numerical format and public perception of foreign immigration growth rates
Source: PLoS One. 2024 Oct 2;19(10):e0310382. doi: 10.1371/journal.pone.0310382 (PMC11446429; doi:10.1371/journal.pone.0310382)
Supplement: S2 Table — (DOCX) [file pone.0310382.s002.docx]

# Appendix S2

**Table S2.** Items used in the survey to measure cultural worldviews.

| Cultural Worldviews |
| --- |
| IINTRSTS. The government interferes far too much in our everyday lives.[I]  CHARM. Sometimes government needs to make laws that keep people from hurting themselves.[C]  IPROTECT. It’s not the government’s business to try to protect people from themselves. [I]  IPRIVACY. The government should stop telling people how to live their lives.[I]  CPROTECT. The government should do more to advance society’s goals, even if that means limiting the freedom and choices of individuals.[C]  CLIMCHOI. Government should put limits on the choices individuals can make so they don’t get in the way of what’s good for society.[C]  HEQUAL. We have gone too far in pushing equal rights in this country.[H]  EWEALTH. Our society would be better off if the distribution of wealth was more equal.[E]  ERADEQ. We need to dramatically reduce inequalities between the rich and the poor, whites and people of color, and men and women.[E]  EDISCRIM. Discrimination against minorities is still a very serious problem in our society.[E]  HREVDIS2. It seems like blacks, women, homosexuals and other groups don’t want equal rights, they want special rights just for them.[H]  HFEMININ. Society as a whole has become too soft and feminine.[H] |

[I] Individualistic; [C] Communitarian; [H] Hierarchical; [E] Egalitarian
