# Supplementary material for: Numerical format and public perception of foreign immigration growth rates
Source: PLoS One. 2024 Oct 2;19(10):e0310382. doi: 10.1371/journal.pone.0310382 (PMC11446429; doi:10.1371/journal.pone.0310382)
Supplement: S3 Table — (DOCX) [file pone.0310382.s003.docx]

# Appendix S3

**Table S3.** Items used in the survey to measure the perception of immigration.

| Perception of Immigration |
| --- |
| Q1. Immigrants improve Italian society by bringing new ideas and cultures.^1^*  Q2. Italian culture is generally undermined by immigrants.^1^*  Q3. Immigrants increase crime rates.^1^*  Q4. Immigrants are generally good for Italian's economy.^1^*  Q5. Immigrants take jobs away from people who were born in Italy.^1^*  Q6. They should be entitled to have their children continue to qualify as Italian citizens if born in Italy.^1^*  Q7. Legal immigrants to Italy who are not citizens should have the same rights as Italian citizens.^1^*  Q8. The government spends too much money assisting immigrants.^1^*  Q9. Refugees should be allowed to stay in Italy.^1^*  Q10. Do you think the number of immigrants to Italy nowadays should be:^2^*  Q11. In general, how risky do you consider the immigration phenomenon to be to Italian society as a whole?^3^  Q12. In general, how beneficial do you consider the immigration phenomenon to be to Italian society as a whole?^3^  Q13. Thinking about the immigration phenomenon in Italy makes you feel __________ emotions:^4^ |
| ^1^ Extremely agree, agree, neither agree nor disagree, disagree, extremely disagree.  ^2^ Increased a lot, increased a little, remain the same as it is, reduced a little, reduced a lot.  ^3^ Extremely risky/beneficial, very risky/beneficial, fairly risky/beneficial, little risky/beneficial, not at all risky/beneficial.  ^4^ Very negative, a little negative, no emotion, a little positive, very positive. |

*Items from the 1974/2014 General Social Survey (Smith *et al.*, 2017)
