# Supplementary material for: Numerical format and public perception of foreign immigration growth rates
Source: PLoS One. 2024 Oct 2;19(10):e0310382. doi: 10.1371/journal.pone.0310382 (PMC11446429; doi:10.1371/journal.pone.0310382)
Supplement: S5 Table — (DOCX) [file pone.0310382.s005.docx]

# Appendix S5

**Table S5.** Items used in the survey to measure science literacy.

| Science Literacy |
| --- |
| Q1. The center of the Earth is very hot.  Q2. All radioactivity is man-made.  Q3. It is the father’s gene that decides whether the baby is a boy or a girl.  Q4. Lasers work by focusing sound waves.  Q5. Electrons are smaller than atoms.  Q6. Antibiotics kill viruses as well as bacteria.  Q7. The continents on which we live have been moving their locations for millions of years and will continue to move in the future.  Q8. It is the Earth that goes around the Sun.  Q9. According to the theory of evolution, human beings, as we know them today, developed from earlier species of animals.  Q10. According to astronomers, the universe began with a big explosion. |
