# Supplementary material for: Numerical format and public perception of foreign immigration growth rates
Source: PLoS One. 2024 Oct 2;19(10):e0310382. doi: 10.1371/journal.pone.0310382 (PMC11446429; doi:10.1371/journal.pone.0310382)
Supplement: S1 Appendix — (DOCX) [file pone.0310382.s007.docx]

**Appendix S7**

**Choice of the cutoff**

**Introduction**

In this Appendix, the most relevant results concerning the choice of cutoff for the variable *Perception of Immigration* are reported. Note that the variable is organized on a 5 level Likert scale, defined as: very low (1), low (2), neither high nor low (3), high (4), very high (5). The different considered cutoffs are: 5 *vs*. 1-4, 4-5 *vs*. 1-3, and 3-5 *vs*. 1-2. For each analysis, it is shown: i) the unadjusted odds ratio (UOR); ii) the adjusted odds ratio (AOR), in order to consider, the confounding variables; iii) the associated 95% confidence intervals.

**Comparison 5 *vs* 1-4**

In this case, the cutoff is defined to have on one side the last category (very high) and on the other the remaining four.

**## Unadjusted OR 2.5% 97.5%**

**## 2.076805 1.398210 3.113695**

**## Adjusted OR 2.5 % 97.5 %**

**## 3.168620 1.956175 5.239353**

The UOR is approximately equal to 2. It differs from the AOR, which is greater than 3. So, considering the confounding variables amplifies the effect size.

**Comparison 4-5 vs 1-3**

This cutoff is the one chosen to be used in the paper. The comparison is among the last two levels and the first three.

**## Unadjusted OR 2.5% 97.5%**

**## 2.768788 1.917147 4.026781**

**## Adjusted OR 2.5 % 97.5 %**

**## 4.029641 2.623136 6.285735**

The difference between the UOR and the AOR is evident. This analysis maximizes the effect size.

**Comparison 3-5 vs 1-2**

Lastly, we merge the last three levels and compare them with the first two.

**## Unadjusted OR 2.5% 97.5%**

**## 2.155837 1.160661 4.146425**

**## Adjusted OR 2.5 % 97.5 %**

**## 2.330167 1.222156 4.599129**

The two values here are greater than 2 and very similar to each other. In this analysis the effect size (in particular, the one that considers the confounding variables) is the lowest. However, a value greater than 2 is a relevant value.

**Conclusion**

The UOR values in relation to the cutoff do not show a trend: the effect is maximum with the chosen 4-5 *vs* 1-3, while it is lower with the other two. In addition, our chosen cutoff has allowed us to study the firm position of respondents (“high” and “very high”) versus the other positions, ensuring simultaneously a good balance in numerical terms in the two groups (as can be seen in Table 1 in the paper).

Concluding, the qualitative results do not depend on the cutoff choice. Therefore, whatever the cutoff:

• the format effect is highly significant even if account shall be taken of the confounding variables;

• interactions are not significant;

• the UOR and the AOR have generally values greater than 2 and the AOR is in all cases numerically greater than the UOR.
