# Supplementary material for: Numerical format and public perception of foreign immigration growth rates
Source: PLoS One. 2024 Oct 2;19(10):e0310382. doi: 10.1371/journal.pone.0310382 (PMC11446429; doi:10.1371/journal.pone.0310382)
Supplement: S2 Appendix — (DOCX) [file pone.0310382.s008.docx]

**Appendix S8**

**Ordinal Logistic Regression**

In this Appendix, most relevant results obtained fitting an ordinal logistic regression are reported. Firstly, we have examined only the format variable to estimate its unadjusted effect (without considering potential confounding variables). In the following table, the results are presented.

| Variable | Estimate | Std. Error | *t* value |
| --- | --- | --- | --- |
| Format  1-in-X *vs* Percentage | 0.877 | 0.163 | 5.392 |

Starting from the results presented in the Appendix B, the unadjusted odds ratio (UOR) assume the following values:

- 2.08 when comparing 5 with 1-4;
- 2.77 when comparing 4-5 with 1-3;
- 2.16 when comparing 3-5 with 1-2.

It is possible to note that the UOR value 2.4 (i.e. $exp(0.877)$) of the ordinal logistic model is totally comparable with those obtained with different cutoffs. The estimate of the effect size is, therefore, independent by chosen cutoff and by the type of chosen model.

An analysis considering all the potential confounding variables has also been performed. Three models are compared:

1. a model containing the main effects only of confounding variables;
2. a model that adds the main effect of format;
3. a model that considers also the interactions between format and confounding variables.

The results are reported here. When the format is added, it maintains its highly significant effect (*p*-value equal about 10^-12^). The LRT, with 1 *df*, is 50.931 and has *p*-value < 0.001.

The interactions between the format and the confounding variables are not statistically significant (LRT= 11.458; *df* = 8; *p*-value = 0.177).

Hence, the final model is the one with the main effects of confounding variables and of format. In the following, the summary table.

| Variables | Estimate | Std. Error | *t* value |
| --- | --- | --- | --- |
| Gender Male vs Female | 0.200 | 0.172 | 1.159 |
| Age  36-55 *vs*. 18-35  56-80 *vs*. 18-35 | 0.067  –0.345 | 0.242  0.231 | 0.278  –1.494 |
| Numeracy | –0.188 | 0.091 | –2.053 |
| Economic literacy | 0.014 | 0.042 | 0.342 |
| Scientific literacy | 0.076 | 0.067 | 1.140 |
| Cultural worldviews  HI *vs*. EC | –0.103 | 0.129 | –0.547 |
| Perception of immigration | –1.799 | 0.152 | –11.847 |
| Format  1-in-X *vs* Percentage | 1.221 | 0.175 | 6.967 |

It is possible to compare these results with those obtained with the cutoff chosen and presented in the main text. Some evidences can be summarized as:

- the coefficients magnitudes are the same;
- the variable “*Perception of immigration*” is confirmed highly significant, with an absolute value of the test higher for logistic model;
- the format variable has a coefficient equal to 1.22 (UOR = 3.39) in the ordinal model and a higher value in the logistic model (1.39; UOR = 4.01). In both cases, the effect is highly significant.
