# Supplementary material for: Numerical format and public perception of foreign immigration growth rates
Source: PLoS One. 2024 Oct 2;19(10):e0310382. doi: 10.1371/journal.pone.0310382 (PMC11446429; doi:10.1371/journal.pone.0310382)
Supplement: S3 Appendix — (DOCX) [file pone.0310382.s009.docx]

**Appendix S9**

**Linear regression**

In this Appendix, results obtained performing linear regression models are presented. To implement such models, the dependent variable needs to be considered on a continuous scale (Agresti and Finlay, 2009, chapter 12). First, the mean values of two formats are analysed (the coding of the variable is the same used in the main text).

**## First sample: mean = 3.607004 sd = 0.9422948 n = 257**

**## Second sample: mean = 4.028881 sd = 0.8800711 n = 277**

**##**

**## Variance pooled = 0.8290909 ES = 0.07886154**

**## t = 5.349591 df = 532 p-value = 1.311717e-07**

**## critical t = 1.964433**

**##**

**## alternative hypothesis: true difference in means is not equal to 0**

**## 95 percent confidence interval:**

**## from -0.5767952 to -0.2669588**

The first format shows a mean equal to 4.03, while the second format shows a mean equal to 3.61. This difference is highly significant, with a *p-value*comparable to the one of the logistic model (10^−7^ *vs* 10^−8^) presented in the paper.

Following the analysis proposed in the paper, here the joint effect of the confounding variables (Gender; Age: 3 categories; Numeracy; Economic literacy; Scientific literacy; Cultural worldviews; Perception of immigration) is considered.

As a first analysis, all the variables are considered as linear effects. The estimated linear model contains only the main effect of the confounding variables. The results are very similar to those obtained with the logistic model. The magnitude of the coefficients is quite similar. It is confirmed that the only significant variable is the “*Perception of immigration”*.

In the second instance, in the model has been inserted the main effect of the format. Also, in this case, after having controlled for the confounding variables, the effect exists and is highly significant

(*F* test = 45.933; df = 1; *p* < 0.001).

Before showing the coefficient estimates of the final model, we evaluated the eventual existence of significant interactions with the format. So, we estimated a third linear regression model in which all the interactions between the confounding variables and the format variable have been considered. The ANOVA comparing the full model (containing all the interactions) with the “reduced” model (which contains only the main effects of the considered variables) discards the full model, with a *p-value* higher than 5% (*F* test= 1.750; df = 8; *p*–value = 0.084).

Finally, in the following table is shown the final model.

| Variables | Estimate | Std. Error | *t* value | *p*–value |
| --- | --- | --- | --- | --- |
| (Intercept) | 5.571 | 0.277 | 20.124 | 0.000 |
| Gender  Male vs Female | 0.060 | 0.070 | 0.862 | 0.389 |
| Age  36-55 *vs*. 18-35  56-80 *vs*. 18-35 | 0.055  –0.091 | 0.097  0.093 | 0.568  –0.982 | 0.570  0.326 |
| Numeracy | –0.062 | 0.037 | –1.671 | 0.095 |
| Economic literacy | 0.008 | 0.017 | 0.486 | 0.627 |
| Scientific literacy | 0.020 | 0.027 | 0.741 | 0.459 |
| Cultural worldviews  HI *vs*. EC | –0.038 | 0.078 | –0.491 | 0.623 |
| Perception of immigration | –0.636 | 0.051 | –12.542 | 0.000 |
| Format  1-in-X *vs* Percentage | 0.459 | 0.068 | 6.777 | 0.000 |

The format effect is not modified by any of the other variables and remains highly significant. Very interesting is that the difference between the means adjusted for the confounding variables remains substantially the same obtained with the basic analysis (the comparison made by t test) and, numerically, it is even higher. A similar result is obtained with a logistic model. None of the confounding variables is associated with the response variable, except the “*Perception of immigration*”, which is negatively associated.

A difference of 0.42 (i.e. 4.03 – 3.61) between the “raw” means of the two groups corresponds to a Cohen’s *d* (Cohen, 1992) of 0.46 (taking into account the pooled standard deviation). The value of Cohen’s *d* is even greater (0.59) when the effect of confounding variables is considered.

**References**

Agresti A., Finlay B. (2009) *Statistical Methods for the Social Science*, Fourth edition, Pearson Prentice Hall, Upper Saddle River, New Jersey.

Cohen J. (1992) A power primer. *Psychological Bulletin*, 112 (1), 155-159.
